# Performance Optimization for Bionic Robotic Dolphin with Active Variable Stiffness Control

**DOI:** 10.3390/biomimetics8070545

**Published:** 2023-11-13

**Authors:** Di Chen, Yan Xiong, Bo Wang, Ru Tong, Yan Meng, Junzhi Yu

**Affiliations:** 1 State Key Laboratory for Turbulence and Complex Systems, Department of Advanced Manufacturing and Robotics, College of Engineering, Peking University, Beijing 100871, China; 2 Laboratory of Cognitive and Decision Intelligence for Complex System, Institute of Automation, Chinese Academy of Sciences, Beijing 100190, China; tongru2019@ia.ac.cn; 3 Science and Technology on Integrated Information System Laboratory, Institute of Software, Chinese Academy of Sciences, Beijing 100190, China

**Keywords:** robotic dolphin, torque control, variable stiffness mechanism, performance optimization

## Abstract

Aquatic animals such as fish and cetaceans can actively modulate their body stiffness with muscle to achieve excellent swimming performance under different situations. However, it is still challenging for a robotic swimmer with bionic propulsion mode to dynamically adjust its body stiffness to improve the swimming speed due to the difficulties in designing an effective stiffness adjustment structure. In this paper, based on the special torque mode of a motor, we propose an active variable stiffness control method for a robotic dolphin to pursue better swimming speed. Different from a variable stiffness structure design, a torque control strategy for the caudal motor is employed to imitate the physical property of a torsion spring to act as the variable stiffness component. In addition, we also establish a dynamic model with the Lagrangian method to explore the variable stiffness mechanism. Extensive experiments have validated the dynamic model, and then the relationships between frequency and stiffness on swimming performance are presented. More importantly, through integrating the dynamic model and torque actuation mode-based variable stiffness mechanism, the online performance optimization scheme can be easily realized, providing valuable guidance in coordinating system parameters. Finally, experiments have demonstrated the stiffness adjustment capability of the caudal joint, validating the effectiveness of the proposed control method. The results also reveal that stiffness plays an essential role in swimming motion, and the active stiffness adjustment can significantly contribute to performance improvement in both speed and efficiency. Namely, with the adjustment of stiffness, the maximum speed of our robotic dolphin achieves up to 1.12 body length per second (BL/s) at 2.88 Hz increasing by 0.44 BL/s. Additionally, the efficiency is also improved by 37%. The conducted works will offer some new insights into the stiffness adjustment of robotic swimmers for better swimming performance.

## 1. Introduction

Over the eons of evolutionary time, marine animals have adapted perfectly to the complex aquatic environment with their unique body structure and motion mechanism. Thereinto, fish and some cetacean animals with undulatory propulsion mode, such as Tuna and dolphin, are among the best candidates in terms of underwater swimming performance including speed, maneuverability, efficiency, and stealthiness, which provides extensive inspiration for the development of underwater robots [1,2,3,4,5]. Over the last couple of decades, bionic robotic fishes or robotic dolphins have attracted extensive attention from researchers and a large number of prototypes have been developed and tentatively used in marine environment monitoring and protection [6,7], marine animals supervision [8], and deep-sea exploration [9]. However, the swimming performance of these bionic robots is still inferior to ocean animals, which restricts their further applications. By learning from ocean biology including propulsion mode and structural characteristics in depth, the performance of underwater robots can be improved significantly to adapt to more extreme environments.

From the perspective of motion mechanism, underwater swimming robots are usually designed with multi-joint rigid discrete links to pursue better swimming performance. The optimization control of joints was conducted to imitate the propulsion mode with the fish body wave model. For example, Yu et al. combined the dynamic model and particle swarm optimization (PSO) algorithm to optimize the central pattern generators (CPG) parameters for an enhanced performance [10]. Hong et al. proposed a deep reinforcement learning method to optimize the body wave of a multi-joint robotic fish for better swimming speed and efficiency [11]. In terms of the body structure, the characteristic of flexibility is emerging as one of the focuses of swimming robots. Many investigations indicate that adding some flexibility into the body, joints, and fins of robotic fish can enhance the performance significantly [12]. For instance, Zou et al. used spring steels as two flexible joints of fishtail and analyzed the influence of both joint stiffness and frequency on swimming speed. An optimal swimming speed of 0.3 body length per second (BL/s) was obtained at 2.5 Hz [13]. Liao et al. proposed a wire-driven elastic robotic fish that can imitate continuous fish-like undulation propulsion and reach a speed of 0.58 m/s (1.04 BL/s) [14]. White et al. designed a passive peduncle joint with a lateral keel for Tunabot which achieves a speed of 4.0 BL/s at 15 Hz [15]. Furthermore, Tunabot Flex was developed with three body flexibility configurations. By analyzing the number of compliant joints, Tunabot Flex gains a speed of 4.6 BL/s at 8.0 Hz [16]. Chen et al. designed a compliant passive joint with two torsion springs for a multi-joint robotic fish and explored the swimming performance under different control parameters and joint stiffness to realize the simultaneous improvement of speed and efficiency at higher frequency [17]. Then, by integrating the high-frequency oscillation and compliant joint, a miniature robotic fish was developed to achieve a speed of 3.8 BL/s at a frequency of 12.8 Hz [18]. In addition, the stiffness of the joint was optimized with experiments, and a new robot achieves a speed of 7.1 BL/s at a frequency of 15 Hz [19]. Jiang et al. developed a fish robot with tension structures to explore the swimming mechanism of stiffness distribution. Different stiffness distributions were configured offline to verify the effectiveness in improving swimming speed with a maximum swimming velocity of 0.87 BL/s [20]. As mentioned above, benefiting from the flexible components, the robotic fish have obtained excellent swimming speed. However, the optimal stiffness of the flexible structure is usually determined by experiments and cannot be adjusted online, which limits the further improvement of performance in a broad range of situations.

In nature, the body of fish or dolphins is actuated by continuous flexible muscles that can regulate the body stiffness actively to achieve outstanding swimming performance at different locomotions. Inspired by this phenomenon, many researchers have been devoted to the investigation of variable stiffness mechanisms for better swimming performance [21]. Park et al. proposed a variable-stiffness flapping mechanism with the endoskeleton structure of dolphin [22,23]. It was made by bonding alternating rigid segments and compliant segments in series. The stiffness can be changed by compressing the compliant segments to maximize the thrust. Similarly, Zhu et al. created a variable stiffness tail that combines a hard silicone cylinder with several elastic balls [24]. By squeezing the inner balls, the stiffness of the tail can be increased by 26.46%. For the complex structure, they have not been integrated into a robotic fish platform. Jusufi et al. designed a tethered soft robotic fish with pneumatic actuators attaching to a flexible foil [25]. By changing the pressure, the body stiffness can be adjusted and a maximum speed of 0.13 m/s was obtained. Zhong et al. explore the stiffness modulation mechanism of fish to achieve efficient swimming [26]. A tail with a tunable stiffness structure is designed for a tethered robotic tuna. Experiments suggest that tuning stiffness can double the swimming efficiency. Li et al. constructed a soft robotic fish with a variable stiffness decoupled mechanism to explore the relationship between stiffness and swimming performance [27]. The stiffness can be adjusted online to improve the swimming speed at different frequencies. The maximum speed is about 0.54 BL/s at 2.4 Hz. Qiu et al. developed a tendon-driven robotic fish with a variable stiffness tail and realized a speed improvement [28]. Sun et al. proposed a fish-tail-inspired method to optimize the stress distribution of a tendon-driven continuum robot to enhance its performance [29]. The variable stiffness mechanism has great potential to enhance performance, but the robotic fish has yet to benefit from this significantly. The complicated variable stiffness structures with large sizes cause difficulty in the design of robotic fish platforms. The stiffness variation also causes the shape change lacking flexible control. In addition, the explorations of variable stiffness mechanisms mainly focus on the structure design, and the effects are tested with experiments. Therefore, some new methods of variable stiffness mechanism with numerical optimization methods should be proposed to promote performance improvement.

In this paper, we mainly focus on the performance optimization of a self-propelled robotic dolphin with a torque control-based variable stiffness mechanism. To achieve this goal, a robotic dolphin with powerful dorsoventral propulsion is introduced. Different from the traditional variable stiffness structure, we take advantage of a unique torque mode of the actuation motor to imitate the compliant component in the caudal joint. Namely, according to the properties of torsion springs, a control method is employed to adjust the torque output of the joint at different joint angles. Therefore, the stiffness adjustment of the joint can be also achieved easily by setting different stiffness coefficients. A dynamic model of the robotic dolphin with the active variable stiffness mechanism is built to analyze the influence of control parameters and stiffness on swimming performance, and an online performance optimization scheme is proposed to improve the swimming speed. Finally, extensive experiments have validated the effectiveness of the proposed variable stiffness mechanism on performance improvement.

The rest of this paper is organized as follows. Section 2 briefly presents the mechanical design of our robotic dolphin and the active variable stiffness joint actuated by a motor with torque mode. In Section 3, a dynamic model of the robotic dolphin with a compliant joint is established. In Section 4, the dynamic mode is validated with experiment results, and extensive experiments are conducted to analyze the parameters’ influence on swimming performance. Section 5 gives some detailed discussions. Finally, the conclusions are summarized and future works are presented in Section 6.

## 2. Overview of the Robotic Dolphin with Torque Mode-Based Variable Stiffness Mechanism

### 2.1. Overview of the Robotic Dolphin

**Figure 1:**
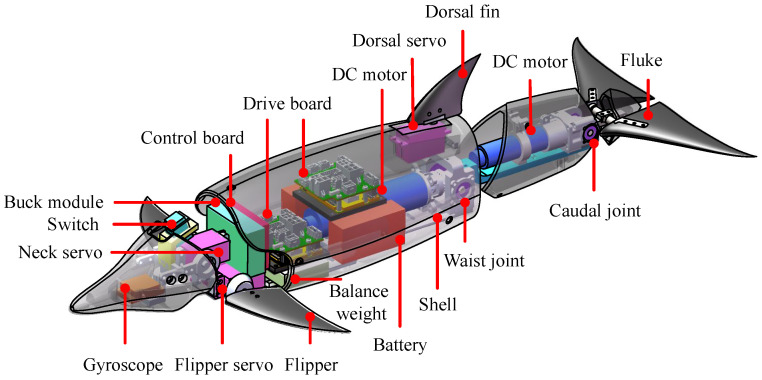
Mechanical design of the bionic robotic dolphin.

Drawing from a dolphin, a novel robotic dolphin has been developed. Figure 1 presents its detailed mechanical design. A well-streamlined body profile is adopted to ensure low hydrodynamic drags. In particular, the dorsoventral propulsion mode with three joints is employed. Namely, a neck joint actuated by a servo motor is integrated to exclusively provide a pitch moment for high-pitch maneuverability. Two principal joints including a waist joint and a caudal joint are configured in the posterior body to provide the powerful undulation propulsion. A pair of flukes connecting the caudal joint generates the main thrust. In addition, to realize pitching motion, a pair of flippers with two degrees of freedom (DOFs) is designed in the near of neck joint. Moreover, for increasing the yaw maneuverability, a dorsal fin actuated by a servo motor is also designed. To emulate the powerful dorsoventral propulsion pattern of a dolphin, two high-performance brushless direct current motors (MAXON EC-4 pole 200 W and 90 W) with gearbox (GP 32 HP, 33:1, GP 22 HP, 24:1) are used for the actuation of waist joint and caudal joint. By integrating the specialized motor controllers (MAXON, EPOS4 Compact 50/15), the motors can implement high-precision position control, velocity control, and torque control. In this paper, we mainly focus on the powerful propulsion with high swimming speed, therefore, the neck joint and flippers are kept still. Various electronic components including sensors, a control board, power supplies, and communication modules are integrated into the robot. Moreover, lightweight and high-strength materials are selected to build the prototype of our robotic dolphin. To enhance the stability of the prototype on the water surface, we added some balance weight (about 0.5 kg) in the appropriate position. The final prototype is about 3.91 kg with a length of 66 cm. The detailed configurations are tabulated in Table 1.

### 2.2. Torque Control-Based Variable Stiffness Mechanism

**Figure 2:**
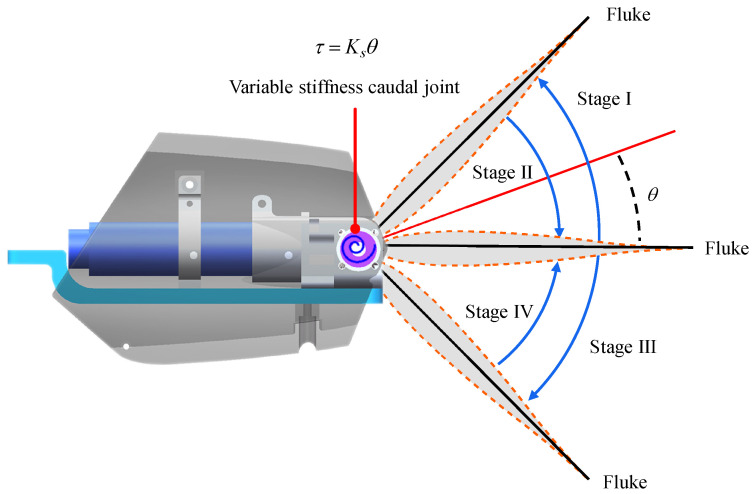
Illustration of torque actuation mode for imitating a torsion spring.

As for the developed robotic dolphin, the waist joint and caudal joint are responsible for the dolphin-like propulsion. The motor of the waist joint plays a key role in overcoming the drag, in this paper, the waist joint is actively actuated by position control mode (profile position mode, PPM) to oscillate in reciprocal sinusoidal form. To imitate the flexible propulsion, the caudal joint is chosen as a compliant joint. A flexible component in a swimming robot usually performs as a passive bent, which provides a torque to keep its original shape. Inspired by this, by controlling the output torque of the caudal motor, the caudal joint can imitate a compliant joint. Owing to the excellent effect in speed enhancing [19], the caudal joint is expected to be similar to a flexible passive joint that embeds torsion springs. Then, the oscillation of flukes with a compliant caudal joint can be illustrated in Figure 2. In the stroke of I and III, the flukes oscillate outward under the action of hydrodynamic force and the motion of the front link, and then a torque generated by flexible component trends to resist the rotation. In the stroke of II and IV, the flukes rotate backward under the promotion of output torque. In brief, a torque should be generated to attempt to keep the fluke in the initial position. According to Hooke’s law of torsion spring, the torque is proportional to the rotation angle of the joint, namely, $\tau =K_{s}\theta$ where τ means the torque generated by the flexible component, $K_{s}$ indicates the stiffness coefficient, and θ is the joint angle.

**Figure 3:**
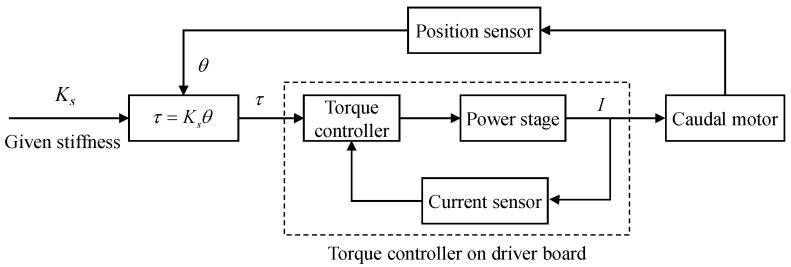
Framework diagram of the torque control strategy for the caudal joint.

To imitate the torsion spring’s flexible physical property, we can employ the cycle synchronous torque mode (CST) of the motor driver to control the output torque [30]. As illustrated in Figure 3, a torque control strategy is proposed for the caudal joint to achieve the flexible joint imitation with the capability of active variable stiffness mechanism. Specifically, by setting a desired stiffness coefficient $K_{s}$ and measuring the joint angle θ of the caudal joint with the help of an encoder, the target torque τ can be obtained in time, then, the motor driver can easily control the torque output of the motor in a closed loop. Concretely, the internal current loop mode is used to adjust the torque output of the motor. In the oscillation of the flukes, the motor performs a variable torque control to imitate the passive actuation feather of flexible components. It is worth noting that the torque controller is provided by the Maxon motor manufacturer and integrated into the motor drive board. We only need to provide the target torque value to the drive board. Finally, by changing the relationship between the output torque τ and joint angle θ, namely, the stiffness coefficient $K_{s}$, the variable stiffness mechanism of the caudal joint can be performed easily.

## 3. Dynamic Modeling of Robotic Dolphin

To optimize the stiffness of the caudal joint at different undulation frequencies, a dynamic model is established. With the specific mechanical design, the developed robotic dolphin is not proficient in yaw maneuvers. In addition, the main goal is to explore the optimal stiffness with frequency, therefore, only the locomotion in the vertical plane is considered. In this study, the caudal joint is taken as a compliant joint with a torque control strategy to explore the variable stiffness mechanism of the robotic dolphin, therefore, the joint angle is unknown and the Lagrangian dynamic approach is employed [17,31].

### 3.1. Kinematic Analysis

**Figure 4:**
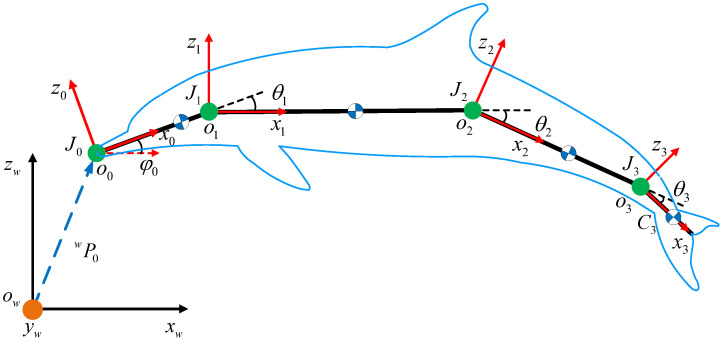
Illustration of coordination frames and notations.

As illustrated in Figure 4, coordinate frames and notations are defined to clarify the dynamic modeling. The robotic dolphin is simplified as a four-link structure. The inertial frame and link frames are defined as $C_{w}=o_{w}-x_{w}y_{w}z_{w}$ and $C_{i}=o_{i}-x_{i}y_{i}z_{i},\left(i=0,1,2,3\right)$, respectively. The plane $x_{w}o_{w}z_{w}$ coinciding with plane $x_{i}o_{i}z_{i},\left(i=0,1,2,3\right)$ is parallel to the vertical plane. $\varphi_{0}$ denotes the pitch angle of the robotic dolphin. $\theta_{i}$ represents the joint angle. Then, $\varphi_{i}=\varphi_{0}+\sum_{j=1}^{i}\theta_{j},\left(i=1,2,3\right)$ represents the angle between *i*th link and axis $o_{w}x_{w}$.

According to the definitions of frames and notations, the kinematics can be derived easily. The position vector of an arbitrary point in link $L_{i}$ can be calculated as

(1) $$r_{i}\left(l\right)={}^{w}P_{0}+{}^{w}R_{0}{}^{0}P_{i}\left(l\right),\left(i=0,1,2,3\right),$$

where ${}^{w}P_{0}$ means the position vector of $J_{0}$ in inertial frame, ${}^{w}R_{0}$ is the rotation matrix of the body frame $C_{0}$ with respect to the inertial frame, and ${}^{0}P_{i}\left(l\right)$ indicates the position vector expressed in $C_{0}$.

The translational velocity and angular velocity can be obtained by taking the time derivative of the position vector and angle vector:

(2) $$\left\{\begin{array}{c} {}^{w}V_{i}={\dot{r}}_{i}={}^{w}{\dot{P}}_{i}+{}^{w}{\dot{R}}_{0}{}^{0}P_{i}\left(l\right)+{}^{w}R_{0}{}^{0}{\dot{P}}_{i}\left(l\right)\\ {}^{w}\Omega_{i}={\left[0,\dot{\varphi_{i}},0\right]}^{T} \end{array}\right.,\left(i=0,1,2,3\right).$$

### 3.2. Lagrangian Dynamic Modeling

In the design of the robotic dolphin, the caudal joint is utilized to imitate a compliant joint with the capability of variable stiffness. Concretely, in the proposed torque control strategy, a torque mode is employed to actuate the caudal joint which can be regarded as a passive joint with a torsion spring. To describe the robot system completely, the generalized coordinates are selected as $q={\left[x_{0},z_{0},\varphi_{0},\varphi_{3}\right]}^{T}$, and then, the generalized velocities can be calculated as $\dot{q}={\left[{\dot{x}}_{0},{\dot{z}}_{0},{\dot{\varphi }}_{0},{\dot{\varphi }}_{3}\right]}^{T}$. As for the robotic dolphin, the kinetic energy $T(q,\dot{q})$ can be presented as follows:

(3) $$T\left(q,\dot{q}\right)=\sum_{i=0}^{3}\frac{1}{2}{}^{w}v_{i}^{T}M_{i}{}^{w}v_{i}+\sum_{i=0}^{3}\frac{1}{2}{}^{w}\omega_{i}^{T}I_{i}{}^{w}\omega_{i},$$

where $M_{i}$ and $I_{i}$ denote the mass matrix and inertia matrix of link $L_{i}$, respectively. The added mass effects are also considered, and can be calculated as follows:

(4) $$M_{a,i}=\int_{0}^{l_{i}}\frac{1}{4}c_{m,i}\rho \pi h_{i}{\left(l\right)}^{2}dl,$$

where $l_{i}$ is the length of *i*th link, $c_{m,i}$ indicates the added mass coefficient, ρ denotes the density of water, and $h_{i}\left(l\right)$ represents the immersed height.

As for the caudal joint, a torque control strategy is proposed to perform passive rotation with a torsion spring-like compliant component. Namely, the variable torque acting on the caudal joint can be expressed in the following form:

(5) $$\tau_{c}=-K_{s}\theta_{3},$$

where $\theta_{3}$ is the caudal joint angle.

Ultimately, the Lagrange equations can be expressed as follows:

(6) $$\left\{\begin{array}{c} \;\frac{d}{dt}\frac{\partial L}{\partial {\dot{x}}_{0}}-\frac{\partial L}{\partial x_{0}}=F_{x}\\ \;\frac{d}{dt}\frac{\partial L}{\partial {\dot{z}}_{0}}-\frac{\partial L}{\partial z_{0}}=F_{z}\\ \;\frac{d}{dt}\frac{\partial L}{\partial {\dot{\varphi }}_{0}}-\frac{\partial L}{\partial \varphi_{0}}=\tau_{0}\\ \;\frac{d}{dt}\frac{\partial L}{\partial {\dot{\varphi }}_{3}}-\frac{\partial L}{\partial \varphi_{3}}=\tau_{3} \end{array},\right.$$

where *L* is the Lagrangian, $F_{x}$ and $F_{z}$ indicate the generalized forces in the direction of $x_{w}$ and $z_{w}$, respectively, $\tau_{0}$ and $\tau_{3}$ denote the generalized moment relating to $J_{0}$ and $J_{3}$ in $y_{w}$, respectively.

### 3.3. Hydrodynamic Analysis

The free-swimming motion is achieved through the interaction between the robotic dolphin and the surrounding fluid. Therefore, the generalized forces are produced by the hydrodynamic forces. In this paper, for the structure characteristics of each link, the Morrison equation and lift/drag model are utilized to evaluate the hydrodynamic forces [32,33], respectively.

As for the body, the Morrison equation where the hydrodynamic forces include added mass forces and drag forces is adopted [32]. The added mass forces in the inertial frame can be calculated as follows:

(7) $$f_{a,i}\left(l\right)=-m_{a,i}\left(l\right){\ddot{r}}_{i}\left(l\right),$$

where $f_{a,i}\left(l\right)$ is the added mass force acting on the per unit length of the link and $m_{a,i}\left(l\right)$ is the added mass in the corresponding location of the slice.

The drag force exerted on the per unit length of the link is generated by the pressure difference and friction viscosity, which is given as follows:

(8) $${}^{i}f_{d,i}\left(l\right)=-\frac{1}{2}\rho \left[\begin{array}{c} c_{f,i}p_{i}\left(l\right)\left|v_{x,i}\left(l\right)\right|v_{x,i}\left(l\right)\\ c_{d,i}h_{i}\left(l\right)\left|v_{y,i}\left(l\right)\right|v_{y,i}\left(l\right)\\ 0 \end{array}\right],$$

where $c_{f,i}$ and $c_{d,i}$ mean the dimensionless friction and drag coefficients, $p_{i}\left(l\right)$ indicates the perimeter of the cross section, $v_{x,i}\left(l\right)$ and $v_{y,i}\left(l\right)$ are the velocity components, respectively. Then, we can obtain the hydrodynamic forces exerted on the *i*th link by integrating the forces along $l_{i}$.

Additionally, the lift and drag model is employed to calculate the hydrodynamic forces acting on the flukes with airfoil profile and given as below.

(9) $$\left\{\begin{array}{c} \;F_{L}=\frac{1}{2}\rho C_{l}\left(\alpha \right)A_{3}v_{3}^{2}\\ \;F_{D}=\frac{1}{2}\rho C_{d}\left(\alpha \right)A_{3}v_{3}^{2} \end{array},\right.$$

where $C_{l}\left(\alpha \right)$ and $C_{d}\left(\alpha \right)$ are the lift coefficient and drag coefficient, respectively, α is the angle of attack, $A_{3}$ is the wetted area of flukes, and $v_{3}$ is the velocity of flukes.

Finally, the generalized forces and moments can be calculated as follows:

(10) $$\left\{\begin{array}{c} \;F_{x}=\sum_{i=0}^{2}F_{a,i,x}+\sum_{i=0}^{2}F_{d,i,x}+{}^{w}F_{3,x}\\ \;F_{z}=\sum_{i=0}^{2}F_{a,i,z}+\sum_{i=0}^{2}F_{d,i,z}+{}^{w}F_{3,z}\\ \;\tau_{0}=\sum_{i=0}^{2}\tau_{a,i,0}+\sum_{i=0}^{2}\tau_{d,i,0}+{}^{w}\tau_{3,0}\\ \;\tau_{3}=\sum_{i=0}^{2}\tau_{a,i,3}+\sum_{i=0}^{2}\tau_{d,i,3}+{}^{w}\tau_{3,3}+\tau_{c} \end{array},\right.$$

where $F_{a,i,x}$ and $F_{a,i,z}$ are the added mass forces of *i*th link in the direction of $x_{w}$ and $z_{w}$, respectively, $F_{d,i,x}$ and $F_{d,i,z}$ are the drag forces of *i*th link in the direction of $x_{w}$ and $z_{w}$, respectively, ${}^{w}F_{3,x}$ and ${}^{w}F_{3,z}$ are the lift/drag force of flukes in direction of $x_{w}$ and $z_{w}$, respectively, $\tau_{a,i,0}$ and $\tau_{a,i,3}$ are torques generated by the added mass forces of *i*th link and relating to $J_{0}$ and $J_{3}$ in $y_{w}$, respectively, $\tau_{d,i,0}$ and $\tau_{d,i,3}$ are the drag forces generated by the drag forces of *i*th link and relating to $J_{0}$ and $J_{3}$ in $y_{w}$, respectively, ${}^{w}\tau_{3,0}$ and ${}^{w}\tau_{3,3}$ denote the torques generated by lift/drag forces of flukes and relating to $J_{0}$ and $J_{3}$ in $y_{w}$, respectively. By integrating these equations, the robotic dolphin can be described, and the locomotion states such as $q={\left[x_{0},z_{0},\varphi_{0},\varphi_{3}\right]}^{T}$ and $\dot{q}={\left[{\dot{x}}_{0},{\dot{z}}_{0},{\dot{\varphi }}_{0},{\dot{\varphi }}_{3}\right]}^{T}$ can be estimated with appropriate dynamic parameters.

## 4. Experiments and Stiffness Optimization

### 4.1. Parameters Identification

In the established dynamic model, many parameters including physical parameters and hydrodynamic parameters should be identified to simulate the swimming motion. By using the software of SolidWorks, we can directly measure or calculate the physical parameters of the robotic dolphin, as tabulated in Table 2. For the calculation simplification of hydrodynamic forces and the irregular shape of the robotic dolphin, the hydrodynamic parameters are difficult to determine directly. Therefore, in this study, we attempt to utilize the experiment results to estimate a set of parameters [34]. Moreover, to simplify the dynamic model, these coefficients are assumed to be constants.

To identify the hydrodynamic parameters of the built dynamic model, some experiments were conducted in an indoor pool whose size is 5 m in length, 4 m in width, and 1.2 m in depth. During the experiments, the swimming motions of the robotic dolphin can be recorded by a global camera located above the pool. With the help of a customized motion measurement system in our previous work [35], we can obtain the swimming speed by analyzing the recorded videos. To better recognize our robotic dolphin, the colorful labels are attached to the body. In addition, the robotic dolphin maintains a little positive buoyancy to swim on the water surface. In this paper, *V* indicates the speed of robot’s center mass (CM) and can be easily obtained with the velocity $V_{0}={\left[{\dot{x}}_{0},0,{\dot{z}}_{0}\right]}^{T}$ of joint $J_{0}$ and the distance $L_{c}=0.4$ m between CM and $J_{0}$.

**Figure 5:**
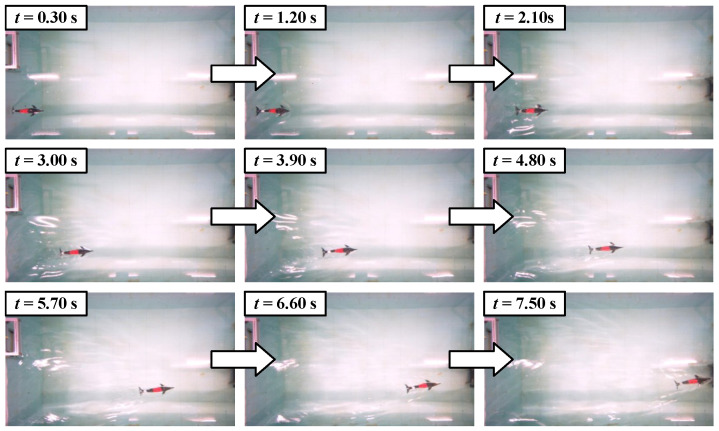
Snapshot sequence of swimming motion used for parameter identification (f= 2.53 Hz, JN1).

**Figure 6:**
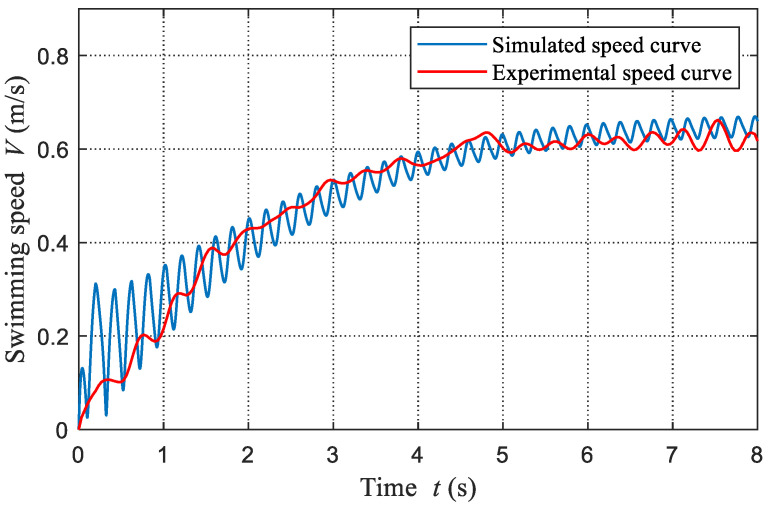
Comparisons between simulated speed curve and experimental speed curve (f= 2.53 Hz, JN1).

The obtained experimental results from a small pool are more accurate and are suitable for the parameter identification of the built dynamic model. In this paper, we primarily concentrate on the swimming speed, therefore, we employ the measured speed as the adopted experimental result. Concretely, the swimming speed curves with a frequency of 2.53 Hz and a stiffness of 0.31 N·m/rad (JN1) are used. Figure 5 presents the snapshot sequence of the corresponding swimming motion. According to some empirical values, we tuned these hydrodynamic parameters manually to fit the experimental speed curve. Ultimately, the identified parameters are acquired and tabulated in Table 3. The comparisons between the experimental speed curve and simulated speed curve are shown in Figure 6. It can be found that these data match well with each other. The experimental speed curve is smoother than the simulated speed curve, which is mainly attributed to the measurement error. 

**Figure 7:**
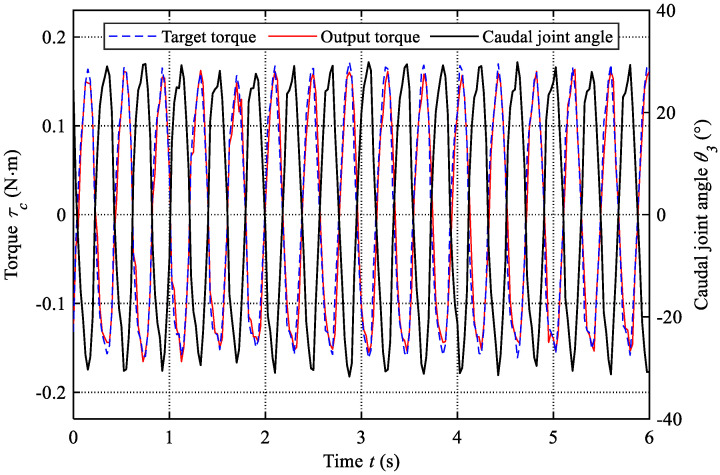
Comparisons between target torque and output torque of the caudal motor (f= 2.53 Hz, JN1).

The torque control of the caudal motor is the foundation of the proposed active variable stiffness mechanism. The drive board provided by the Maxon motor manufacturer is feature-rich and can provide extensive information, such as current, voltage, position, torque, and so on. The output torque of torsion springs is determined by both the joint angle and stiffness coefficient ($\tau =K_{s}\theta$ ), which has the characteristic of passive output. Therefore, during the variable stiffness control, we can calculate the target torque with the measured caudal joint angle and the given stiffness coefficient. In addition, the real output torques are also obtained easily with a sampling frequency of 40 Hz. To evaluate the performance of torque control, we compare the target torque and real output torque of the caudal motor in the experiment with a frequency of 2.53 Hz and a stiffness of 0.31 N·m/rad (JN1). The results are presented in Figure 7. We can find that the output torque can match well with the target torque. Furthermore, the root-mean-square error (RMSE) is also calculated as 0.02 N·m and the normalized RMSE is about 0.06. These results have validated the feasibility of the active variable stiffness mechanism with the torque control mode. In addition, the variation of the caudal joint angle is also presented in Figure 7, which displays the actuation behavior of the caudal joint intuitively.

### 4.2. Experiments Testing

#### 4.2.1. Speed Testing

**Figure 8:**
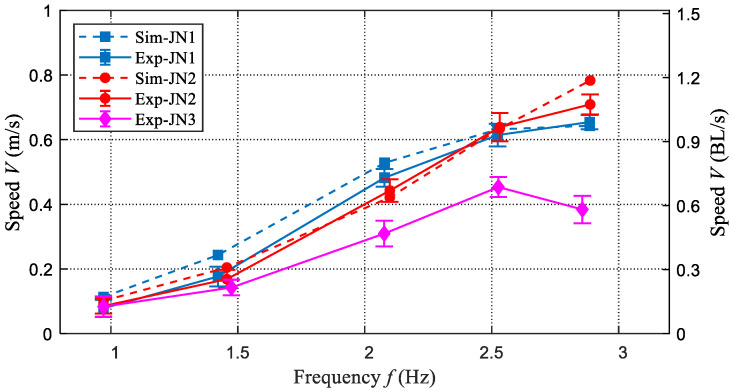
Comparisons between simulated speeds and experimental speeds.

For validating the obtained dynamic model and exploring the influence of parameters, the swimming speeds under different frequencies and stiffness are measured systematically. Due to the limited space of the pool, the frequencies (less 3 Hz) and three stiffness including (JN1, $K_{s}=$ 0.31 N·m/rad, JN2, $K_{s}=$ 0.62 N·m/rad) and rigid joint (JN3) are considered in the conducted experiments. As for these experiments, the oscillation amplitude of the waist joint is about 27${}^{\circ }$. Owing to the action of fluid, the actual actuation frequencies have some differences. The encoder data generated by the waist joint motor is used to calculate the real frequency. In addition, the obtained parameters are integrated into the dynamic model, and the same frequency parameters and stiffness parameters are also used to estimate the steady speed. Finally, we compare the obtained experimental results and the simulated results, as illustrated in Figure 8. The speed RMSEs of JN1 and JN2 are 4.04 cm/s and 3.88 cm/s, respectively. On the whole, these swimming speeds match well with each other, which validates the effectiveness of the established dynamic model to some extent. The large errors are primarily generated at lower frequencies. The reason is that the waves produced by the pool wall have a larger effect on the robot swimming slowly. JN3 represents the caudal joint being locked and the robotic dolphin is changed from a four-link structure to a three-link structure, which is quite different from the built dynamic model. Therefore, the dynamic model is unsuitable to simulate the speed of that case. JN3 is a very special case and is mainly used to reflect the effects of the active variable stiffness mechanism on performance improvement.

**Figure 9:**
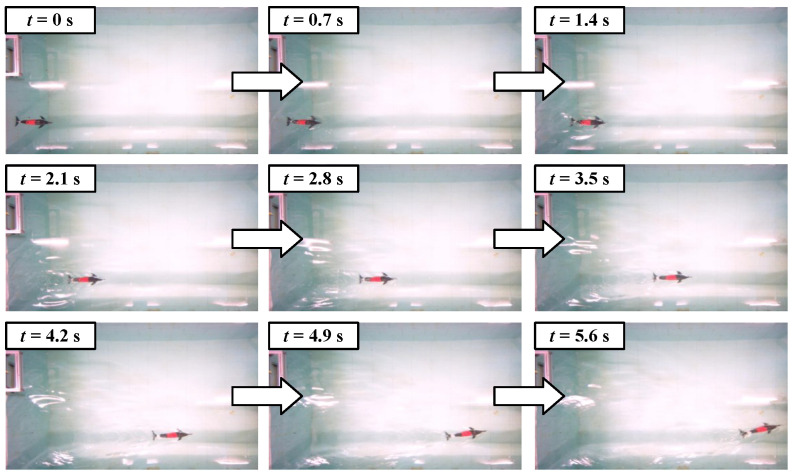
Snapshot sequence of swimming motion with the maximum speed (f= 2.89 Hz, JN2).

As presented in Figure 8, we can find that the frequency and stiffness are essential to the swimming speed. In this study, two stiffness (JN1 and JN2) are chosen to exhibit the capability of actively variable stiffness and explore its effects on swimming speed. The swimming speeds of the two cases all increase with the frequency, and their maximum speeds are 0.71 m/s (1.08 BL/s, JN2) and 0.65 m/s (0.98 BL/s, JN1), respectively. The swimming snapshot sequence of swimming motion with maximum speed is presented in Figure 9. Particularly, the situation of the robotic dolphin with a rigid caudal joint is also considered and the swimming speed curve is presented in Figure 8. In the beginning, the swimming speed increases with the increase of frequency, and the maximum speed reaches up to 0.45 m/s (0.68 BL/s) at a frequency of 2.53 Hz. However, when the frequency exceeds 2.53 Hz, the speed starts to decrease. Namely, the improvement of frequency cannot always ensure the increase of speed. Therefore, the variable stiffness mechanism of flexible joints is crucial for the further improvement of speed. It is worth noting that at the same frequency, the speeds of our robotic dolphin with flexible joints are larger than that of a robotic dolphin with a rigid joint. When the frequency is small, the hydrodynamic forces acting on the flukes are also small, and the caudal joint angle only changes a little. Therefore, the swimming speed is similar to the robotic dolphin with a rigid joint. However, with the increase in frequency, the swimming speed of a robotic dolphin with a flexible joint is gradually greater than that of a robotic dolphin with a rigid joint.

#### 4.2.2. Power Testing

**Figure 10:**
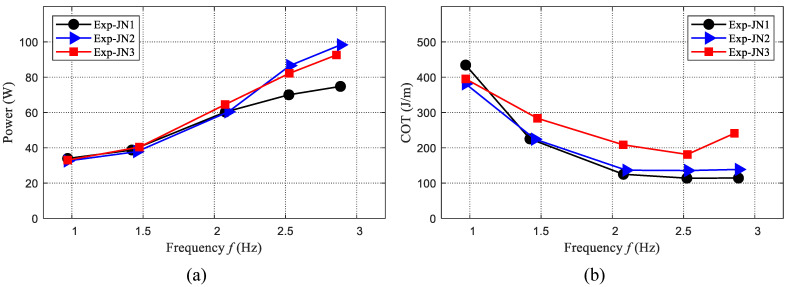
Average power and COT of the robotic dolphin during swimming motion. (**a**) Average power of the robotic dolphin. (**b**) COT of the robotic dolphin.

Besides the swimming speed, efficiency is also important for the robotic dolphin. Therefore, the power of the robotic dolphin during different swimming motions is also measured. The voltage and current are recorded with a sampling frequency of 40 Hz. Finally, the results are presented in Figure 10a. We can find that the power also increases with the frequency. When the frequency is less than 2.1 Hz, the consumed power of the three cases is similar. When the frequency is larger than 2.1 Hz, the case of JN2 consumes the largest power, and the power of the case JN1 is the least.

To evaluate the swimming efficiency, the cost of transport (COT) is selected as an indicator and used to analyze the effect of frequency and stiffness. The COT indicating the consumed power during the robotic dolphin swims a unit distance is defined as the following form:

(11) $$\mathrm{COT}=\frac{P}{V},$$

where *P* is the consumed power and *V* means the swimming speed.

The evaluated COTs are presented in Figure 10b. The COT of the two cases (JN1 and JN2) with a flexible joint is lower than the case (JN3) with a rigid joint, and the case with JN1 is the most efficient. The minimum COT is about 114.3 J/m and is achieved at the maximum speed (1.08 BL/s, JN1). The minimum COT of JN3 is obtained at 2.53 Hz and about 181.2 J/m. The improvement is about 37%. These results suggest that the variable stiffness mechanism can also enhance swimming efficiency. In addition, the maximum speed and the maximum efficiency usually cannot be achieved at the same stiffness.

### 4.3. Performance Optimization

**Figure 11:**
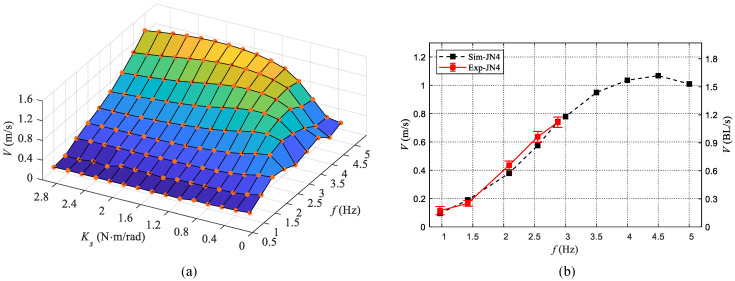
Evaluation and validation of swimming speed versus frequency and stiffness. (**a**) Predicted swimming speed at different frequencies and different stiffness. (**b**) Validation and prediction of swimming speed with a stiffness of 0.8 N·m/rad (JN4).

During swimming motion, the interaction between a robotic dolphin and water is complicated, therefore, the relationship between frequency, stiffness, and swimming speed is challenging to clarify. The means of experiment is usually time-consuming and toilsome. In this study, we take advantage of the validated dynamic model to evaluate the swimming performance in different frequencies and stiffness, which provides a simple and convenient approach. To analyze the influence of frequency and stiffness on swimming motion, we estimate the swimming speed with the established dynamic model. The frequency in the range of [1 Hz, 5 Hz] with an interval of 0.5 Hz and the stiffness in the range of [0.2 N·m/rad, 3.0 N·m/rad] with an interval of 0.2 N·m/rad are considered in the simulation. The results are presented in Figure 11a, from which we can intuitively observe the variation tendency of swimming speed with the variation of frequency and stiffness. Specifically, at each frequency, the effect of stiffness is significant and there exists an optimal stiffness to achieve the maximum speed. When the stiffness is small, the effect of frequency on speed is complicated. However, when the stiffness is larger, the speed will increase with the frequency directly. In addition, we also measured the swimming speed of our robotic dolphin with a stiffness of 0.8 N·m/rad (JN4). The comparisons between experimental data and simulated data are shown in Figure 11b. The maximum speed is about 0.74 m/s (1.12 BL/s, 2.88 Hz) achieving the speed improvement. The RMSE of JN4 is about 3.97 cm/s, validating the model-based prediction to some extent.

**Figure 12:**
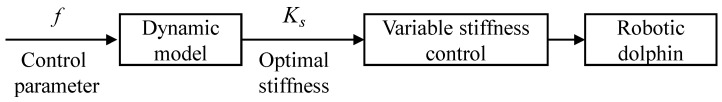
Swimming speed optimization scheme with the online stiffness adjustment.

In nature, biological dolphins can modulate their body stiffness to obtain better swimming performance in time. As for the robotic dolphin with a compliant joint, there also exists an optimal stiffness at a certain condition to achieve maximum swimming speed. The optimal stiffness is mainly determined by oscillation frequency. To explore the proposed variable stiffness mechanism, the optimal coefficient can be estimated with a dynamic model, which helps to achieve high performance including speed and efficiency under a complex environment. Different from the existing variable stiffness mechanism with special structure design, the torque control-based variable stiffness mechanism only requires the parameter adjustment of the motor, which simplifies the performance optimization significantly. In this paper, we mainly focus on pursuing better speed, therefore, by integrating the established dynamic model and the proposed stiffness variable mechanism, we can achieve performance optimization online easily, as shown in Figure 12. With the given actuation frequency, we can utilize the built dynamic model to search for the optimal stiffness $K_{s}$ for maximum speed. Then, $K_{s}$ is set as the target stiffness, and the proposed torque control is adopted to imitate the flexible feather of a torsion spring to achieve the maximum speed. When the control parameters are changed, repeat the above scheme to achieve better swimming speed. Based on the proposed control strategy, the robotic dolphin can easily obtain better swimming speed under different control parameters. In addition, the stiffness optimization for achieving a given speed will be more suitable to perform complex tasks, and is not considered for our robotic dolphin.

## 5. Discussion

In this paper, we adopt the torque control strategy for a caudal motor to imitate the torsion spring characteristics. Namely, the motor outputs a torque that is relevant to the joint angle with a given stiffness coefficient. Therefore, in the whole rotation cycle of flukes, the motor always consumes energy. Compared to the robot with a real torsion spring, our robotic dolphin with a variable stiffness mechanism is deficient in efficiency. A compliant component with a single stiffness can store and release potential energy, where it plays a role of power modulation to improve the speed and efficiency simultaneously. However, to possess the ability of stiffness adjustment, it is inevitable to require extra energy. Additionally, different from the traditional variable structure, the proposed active variable stiffness mechanism is essentially a torque control of a caudal motor that conforms to the conventional system design, therefore, it is easy to design a robotic fish. The torque control accuracy has been validated by the error analysis between target torque and real torque, which can guarantee the reliability of the proposed variable stiffness mechanism. More importantly, in this paper, only a simple situation imitating the stiffness variation characteristic of torsion springs is considered. Based on the proposed active variable stiffness mechanism with torque control of the motor, more torque control strategies, such as changing the stiffness coefficients in one oscillation cycle can be performed to pursue better swimming performance. Through integrating the advantages of the adopted actuation motor in drive mode and the propulsion mechanism of the caudal fin, there is still great exploration space in terms of torque control strategies of the caudal joint to improve the robotic dolphin’s performance, which exhibits strong adaptability.

The interaction between a robotic dolphin and surrounding fluid is extremely complicated. To simplify the analyses, the hydrodynamic forces are estimated with some assumptions, such as fewer fluid factors and hydrodynamic coefficients are considered, which mainly causes the errors between experiment results and simulated results. In this study, only the waist joint with frequency, caudal joint stiffness, and swimming speed are considered. Therefore, based on the dynamic model, we directly present the relationships among these parameters. Towards a multi-joint robotic fish with multi-performances, some intelligent optimization algorithms [36], such as particle swarm optimization, can be integrated into the online optimization scheme.

Finally, we compare the swimming speeds of some robotic fishes with variable stiffness mechanisms, as tabulated in Table 4. It can be seen that only the tethered robotic fish in [26] has a larger speed than our robotic dolphin. However, its frequency is higher than the actuation frequency of our robotic dolphin. The comparison results validate the effectiveness of the proposed variable stiffness mechanism in speed improvement.

## 6. Conclusions

In this paper, based on a special torque drive mode, we have proposed an active variable stiffness control method for a robotic dolphin to improve swimming performance. Inspired by the locomotion characteristic of a flexible joint with torsion springs, a torque control method is proposed for the caudal joint of a robotic dolphin to imitate the variable stiffness mechanism. In addition, a dynamic model for the robotic dolphin is established with the variable stiffness caudal joint, and its effectiveness is also validated. Extensive experiments and model-based simulations are all conducted to explore the influences of key parameters (frequency and stiffness) on swimming performance. These results reveal that the frequency and stiffness play important role in swimming performance. At different actuation frequencies, the stiffness can be optimized to improve swimming speed and efficiency. In addition, the results also demonstrate the capability of variable stiffness, validating the effectiveness of the proposed control method. By adjusting the stiffness of the caudal joint, our robotic dolphin achieves a maximum speed of 1.12 BL/s at a frequency of 2.88 Hz. Finally, combining the built dynamic model and the variable stiffness control method, an online performance optimization scheme for the robotic dolphin is proposed to guide performance improvement.

In future work, we will design a tendon-driven body with tensegrity structure to further enrich the stiffness modulation capability. In addition, we will utilize the technology of computational fluid dynamics (CFD) to further explore the variable stiffness mechanisms of swimming robots in performance improvement. 

## Figures and Tables

**Table 1:** Technical specifications of the robotic dolphin prototype.

Items	Characteristics
Total mass	3.91 kg
Length × Width × Height	0.66 m × 0.11 m × 0.12 m
Power supply	24 V rechargeable batteries
Communication unit	Wireless (RF200, 433 MHz)
Sensor	AHRS, MS5803 01BA
Controller	STM32F407VGT6, 168 MHz
Motor	DC motor × 2, servo motor × 3

**Table 2:** Physical parameters of the robotic dolphin.

Items	Unit	$L_{0}$	$L_{1}$	$L_{2}$	$L_{3}$
$m_{i}$	kg	0.287	2.830	0.662	0.134
$l_{i}$	m	0.134	0.285	0.148	0.093
$I_{r,i}$	kg·m${}^{2}$ ($\times 10^{-3}$)	3.207	79.738	3.742	0.23
$l_{c,i}$	m	–	–	–	0.034
$A_{i}$	m${}^{2}$ ($\times 10^{-3}$)	–	–	–	9.396

${}^{1}$ Note: the value of notation $l_{c,i}$ means the distance between the center of mass of *i*th link and joint $J_{i}$, and $l_{c,3}$ is used in the lift-drag model.

**Table 3:** Hydrodynamic parameters of the robotic dolphin.

Parameters	$c_{m,0}$	$c_{m,1}$	$c_{m,2}$	$c_{f,0}$	$c_{f,1}$	$c_{f,2}$	$c_{d,0}$	$c_{d,1}$	$c_{d,2}$
Value	0.27	0.18	0.26	0.039	0.034	0.015	2.55	4.0	5.8

**Table 4:** Comparisons of existing robotic fishes with variable stiffness mechanism.

Prototype	Frequency (Hz)	Swimming Speed (m/s)	Swimming Speed (BL/s)	Swimming Mode
Robotic fish [25]	0.55	0.13	0.76	tethered
Robotic fish [26]	6.0	0.70	2.0	tethered
Robotic fish [27]	2.4	0.17	0.54	untethered
Robotic fish [28]	2.2	0.47	1.04	untethered
Our robotic dolphin	2.88	0.74	1.12	untethered

## Data Availability

The data generated during the current study are available from the corresponding author on reasonable request.
